# On Some of the Compounds of Urea and a New Method for Determining Common Salt and Urea in Urine

**Published:** 1854-04

**Authors:** J. Liebig


### *On Some of the Compounds of Urea and a New Method for Determining Common Salt and Urea in Urine.*

By J. Liebig.

(Translated for the Examiner.)

From the above mentioned essay, we extract only that part which relates to the quantitative determination of chloride of sodium and urea in urine, and the method requisite for accomplishing it. It is almost needless to mention, that the greatest accuracy is necessary in the measurement of the various fluids, by the burette, which here takes the place of the balance; on this account, we give Liebig's statement in his own words.

*Quantitative Determination of Chlorides in Urine.*—When the chlorine compounds of the alkalies are mixed with the pernitrate of mercury, these salts are decomposed into bichloride of mercury and into a nitrate of the alkaline base.

The pernitrate of mercury immediately produces in a solution of urea, a thick white precipitate, which does not occur with a solution of bichloride of mercury.

Upon these two facts depends the method for the quantitative determination of chlorine in a fluid containing urea and chlorides. If, for instance, we mix a solution of urea and chloride of sodium, and add slowly in small portions, a weak solution of pernitrate of mercury, a white cloudiness will be produced, which immediately disappears upon agitation, the liquid remaining as clear and transparent as before; without the chloride of sodium, however, the solution would have remained permanently cloudy. If we possess therefore a solution of pernitrate of mercury, graduated and determined by means of chloride of sodium, we can by its assistance attain in a very short time the amount of chlorine contained in urine. This graduation is done in the following manner:

Liebig confirms the fact, that when pure transparent rock salt in large pieces is covered with water and allowed to remain, with frequent agitation, for 24 hours, at a temperature of 12° to 24° C. (53.6° to 75.2° F.) the liquid will have dissolved an invariable amount. By evaporating 10 C.C. of this solution, he obtained (as Fuchs and Fehling did also) 3.184 grm. of chloride of sodium. If we now carefully measure out by means of a pipette 20 C.C. of such a solution, (not adding the drops that may adhere to the point of the pipette,) and mix it with 298.4 C.C. of water, then we have 318.4 C.C. of a diluted solution, containing therein $2 \times 3184$ milligrammes of chloride of sodium. In 10 C.C. of this solution are therefore contained 200 milligrammes of chloride of sodium. We then measure by means of a small pipette containing, when filled to a mark on the tube, exactly 10 C.C. of the above mentioned solution, and let it flow into a small beaker glass, then add 3 C.C. of a solution of urea, which in 100 C.C. contains 4 grammes of urea—therefore in 1 C.C. contains 40 milligrammes of urea. The diluted mercurial solution, (the strength of which is to be determined,) is poured into a burette, the height marked at which it stands, and poured drop by drop into the mixed solution of urea and chloride of sodium, which is kept in a state of rotary agitation; as soon as a decided permanent precipitate appears, the test is completed.*

If with 10 C.C. of a solution of chloride of sodium, 7.8 C.C. of a solution of mercury is required for the production of a precipitate, the solution is too concentrated to admit of a correct valuation; it must be diluted with an equal volume of water, and the experiment repeated. If, for instance, 15.5 C.C. of a mercurial solution is required for the production of a cloudiness in 10 C.C. of a solution of urea and chloride of sodium, then to every

155 volumes of this mercurial solution
45 " of water must be added, by which we attain 200 volumes of mercurial solution, of which each 20 C.C. indicates exactly 200 milligrms., or 1 C.C., 10 milligrms. of chloride of sodium. By means of a check experiment the exactness of this measurement can be proved. In mixing 20 C.C. of the test liquid with 10 C.C. of a solution of urea and chloride of sodium, the degree of cloudiness, which is permanently obtained, must be kept in mind. For in the application of this test fluid to the quantitative determination of chloride of sodium if more or less mercurial solution be added, in excess, whereby the cloudiness becomes stronger or weaker, a source of error arises which can easily be avoided by a little practice.†

* No regard need be paid to a mere opalescence of the fluid, as this depends upon the presence of foreign metals, and can easily be recognized as not belonging to the test, since, by the addition of a few drops more of the mercurial solution, the cloudiness does not increase. If the precipitate arises from the compounds of urea, this is not the case, for one drop more of the solution of mercury produces in this case an increased cloudiness.

† The above-described test fluid is intended for those cases in which no foreign salts and no excess of urea are present in the solution; but if employed for the determination of chloride of sodium in urine, there arises a slight error, which makes the amount contained in that fluid appear less than it really is. This error arises from the cloudiness appearing rather earlier, when foreign salts and much urea are present, as in such fluids the precipitate is rather more difficult of solution. A deposit of nitrate of mercury and urea is not produced in the fluid until this is saturated with it; the mercurial solution always contains free nitric acid, which dissolves more of it than water, and this more than a solution of nitrate of urea. Since urine generally contains more urea than was added to the chloride of

To determine now the amount of chlorides in urine, it is necessary, first, to remove the phosphoric acid contained therein. Liebig found that a mixture of one volume of a cold, saturated solution of nitrate of baryta, with two volumes of cold, saturated aqua baryta, was suitable to effect this. We add one or two volumes of this mixture to the urine that is to be examined; the liquid is then filtered from the arising precipitate; it is alkaline from the excess of baryta; this alkaline reaction must be removed by means of nitric acid, though care must be taken to add no more nitric acid than is necessary to restore a slight acid reaction.*

For this experiment then, we must measure in a small pipette, exactly holding the amount, 15 C.C., of this fluid, corresponding to 10 C.C. of urine, pour it into a small beaker glass and mix it, with constant stirring, with the mercurial solution. After the cloudiness appears, the amount of test liquid used can be ascertained from the burette; every C.C. used represents 10 milligrms. of chloride of sodium. Comparative experiment proved that the results obtained in the quantitative determination of chlorine by sodium, in the graduation of the mercurial solution, this urea enters into combination with a part of the free nitric acid of the mercurial salt, forming nitrate of urea, by which the solvent power of the fluid for the precipitate is lessened, the latter appearing earlier, so that less of the test fluid is necessary to produce the reaction. This error may be entirely prevented, if to the 10 C.C. of solution of chloride of sodium, which has been mixed with 3 C.C. of a solution of urea, is added 5 C.C. of a cold saturated solution of sulphate of soda, and the test fluid then graduated. Pernitrate of mercury gives with a solution of sulphate of soda, a yellow pulverulent precipitate of turpeth mineral. If the sulphate of soda contains chloride of sodium, the addition of the nitrate of mercury does not produce a precipitate of turpeth mineral until the whole of the chloride of sodium is converted into bichloride of mercury; on this account the experiment is only affected by the addition of sulphate of soda so far that the free acid of the mercurial salt combines with the sulphate of soda to form an acid salt, whereby the same object is attained as by an excess of urea.

*It is better to acidify with nitric acid the whole filtrate, and not merely the measured out 15 C.C. which is used as a test; a few drops more would not matter in a 100 or more C.C., but added to the small amount employed for the determination, would injure the accuracy of the experiment.

the above described method, were as exact as those by the use of nitrate of silver.

*Quantitative determination of urea in urine.*—The method depends upon the precipitation of the urea by pernitrate of mercury. The explanation is as follows: If a diluted solution of pernitrate of mercury is gradually added to a diluted solution of urea, the free acid of the mixture being neutralized, from time to time, by aqua baryta or a weak solution of the carbonate of soda, a flaky, somewhat spongy, snow-white precipitate, insoluble in water, will be obtained. If the mercurial salt and carbonate of soda are alternately added, as long as a precipitate is formed, the mixture at a certain period will, upon the addition of carbonate of soda, assume a yellowish coloring arising from the basic nitrate of mercury. If the fluid be now filtered it will contain no appreciable amount of urea, for it has all been precipitated. Liebig found that this precipitate always contained one equiv. of urea and four equiv. of oxide of mercury.

If pernitrate of mercury is added to a solution of urea, as long as a precipitate is formed, then the mixture, upon the addition of carbonate of soda remains *white*; but if the original mixture be allowed to stand for a few hours, the nature of the precipitate becomes changed, and is then crystalline, and the six sided leaflets of the combination with three atoms of oxide of mercury are easily recognized, and the clear liquid remaining above the crystals—which was previously precipitated white—now, by the addition of alkalies, gives a yellow precipitate. In the acid liquid, the combination with four atoms of oxide of mercury is therefore transformed into a combination containing less oxide; that is to say, a portion of the oxide enters again into solution. In order to ascertain if the requisite amount of mercurial salt has been added, to produce the compound of urea, with four atoms of oxide of mercury, the solution must be neutralized by carbonate of soda. If a drop of this mixture remains white, when mixed upon a watch glass with a drop of the solution of carbonate of soda, then it can be safely asserted that free urea is present; but if a yellowish film appears upon the surface of the liquid, then the limit is reached, or rather overstepped; it is only necessary now to add a very slight excess of mercurial salt—to indicate that a sufficient amount has been added—for the entire precipitation of the urea. It is evident, that if the amount of mercury in the mercurial solution be known, the amount of urea contained in the liquid can also be known, from the amount of the solution requisite for the precipitation of the urea. Or, if to precipitate a known amount of urea, say 100 milligrms.—we have found it necessary to use a certain volume of mercurial solution—then in a fluid containing an unknown amount of urea, a like volume of the solution, must indicate a corresponding amount of urea. We can calculate the amount of urea from the amount of mercurial solution used; if more or less is requisite—more or less urea is present. After this explanation the method is easily understood. A solution of pernitrate of mercury must be graduated, with which an exact quantitative determination of urea can be made. Liebig, for this purpose, used a solution of which 1 C.C. corresponds precisely to 10 milligrms. of urea. This solution must contain as much oxide of mercury as is necessary to form, with 100 milligrms. of urea, the nitric acid combination with 4 equiv. of oxide of mercury, together with a small excess necessary to indicate the precipitation of the urea, ascertained in the manner above stated. Liebig found that for 100 milligrms. of urea, which, according to the calculation, required 720 milligrms. of oxide of mercury (in the form of the nitrate,) 10 C.C. of the mercurial solution must contain 772 milligrms. of oxide of mercury, in order to produce in diluted fluids a distinct reaction of the mercurial oxide. Each C.C. of mercurial oxide must therefore contain an excess of 5.2 milligrms. of oxide of mercury. The most simple mode of obtaining this fluid is to dissolve pure mercury in pure nitric acid; it must then be warmed and nitric acid added until no more red fumes arise; evaporate it then in the water bath to a syrup consistence, and finally dilute it with so much water, that in 100 C.C. of the diluted solution will be found precisely 7.140 grmms. of mercury. This occurs if we add to 100 grmms. of mercury (changed into nitrate of the oxide) so much water, that the volume of the fluid consists of 1400 C.C. On this account it is therefore desirable not to add the whole calculated amount of water at once, but somewhat less. We then measure 10 C.C. of a solution of urea which contains in 200 C.C. four grmms. of pure urea, and add from a pipette the approximatively diluted mercurial solution, until a few drops of the mixture upon a watch-glass, gives, with a solution of carbonate of soda, a decidedly yellow color.*

Suppose, for this purpose, we have used 19.25 C.C. of mercurial solution, then for each 192.5 C.C. of this solution, we must add 7.5 C.C. of water, and make a new and final test. If, after the addition of 20 C.C. of mercurial solution to 10 C.C. of the above described solution of urea, the appearance of the yellow color is decided, then the mercurial solution can be used for the determination of urea in urine.†

*In order to obtain this solution we dissolve four grms. of pure urea in water, and dilute with water until the volume measures exactly 200CC; by dissolving four grms. urea in 200 C.C. of water, we would obtain 201.75 C.C. of fluid, therefore 1.75 C.C. too much.

† *Observation.*—This test fluid is graduated by a solution of urea, which contains two per cent. of urea; 15 C.C. of this solution requires for the precipitation of the urea, as well as to indicate its completion, 30 C.C. of mercurial solution; we obtain 45 C.C. of mixture, in the whole of which exists $30 \times 5.2 = 156$ milligrms. of oxide of mercury; each C.C. contains about 3.47 milligrms. of oxide of mercury. If the 15 C.C. of the solution of urea contains four per cent. of urea, and 60 C.C. of mercurial solution added, then we have 75 C.C. of mixture, in which are 312 milligrms. of oxide of mercury; in each C.C., 4.16 milligrms., therefore, 0.69 milligrms. of oxide of mercury more than is requisite to produce the original color. We will, therefore, be liable to an error, as experiments have shown, in the analysis of urine, when the amount of urea is increased, whereby the apparent amount of urea will be diminished. In the above stated case we would have added, until the appearance of the original color, not 60 C.C., but only 59.37 C.C. of mercurial solution. To avoid this error, we must, when experimenting upon 15 C.C. of urine, add to every C.C. above 30 of the mercurial solution that has been used for the precipitation of the urea, half the number of C.C. of water, *before* the test is made with carbonate of soda; if, for instance, we use 20 C.C. more, we must add 10 C.C. more of water. It will always be found necessary, after the addition of water, to add a few drops of mercurial solution, to obtain the indication of a complete precipitation. From the same reason, we must, in those cases where the amount of urea is only one per cent., add for 15 C.C. of urine, not 15 C.C., but 15.3 C.C. of mercurial solution. To avoid this mistake, which increases the amount, we must from each 5 C.C. of mercurial solution less than 30, that have been used, subtract 0.1 C.C. If we, therefore, use 25 C.C. of mercurial solution for 15 C.C. of urine, the amount, 249 milligrms., would be expressed by 24.9 C.C. of solution, &c., &c.

For the determination of urea in urine, we must first prepare a mixture of two vols. of aquæ baryta, with one vol. of a solution of nitrate of baryta, both saturated cold; and mix one vol. of this alkaline fluid with two vols. of urine. For this purpose, use a small glass cylinder of the desired size, which must be first filled *twice* with urine, even to overflowing. The mouth of the cylinder must each time be covered with a glass plate, so that the excess may flow off. The same cylinder must be filled in the same manner, *once* with the solution of baryta, and this poured into the urine in a beaker glass; a precipitate arises from the mixture which must be filtered. From the whole mixture we must measure out for each analysis, 15 C.C., which corresponds to 10 C.C. of urine. Into this volume of urine, before neutralizing it, (as is done in the determination of the chlorides,) must be poured from a burette the graduated solution of pernitrate of mercury, continually stirring it, and when no further precipitation (no thickening of the fluid) is perceived, then test it by pouring a few drops with the precipitate from the beaker glass on to a watch glass, and adding to it a few drops of the solution of carbonate of soda, best done from a caoutchouc pipette. If, after a few minutes, the mixture retains its white color, then more mercurial solution must be added to the mixture in the beaker glass, until, by testing with carbonate of soda, a decided yellow color is perceived. Then read off the number of C.C. used, and correct the number obtained according to the contents of urine, in the manner given above. Should the urine contain no chloride of sodium, then every thing has been done for the quantitative determination of the urea. A series of experiments have shown, that if there is from 1—1½ per cent. of chloride of sodium in urine, it exercises, by means of the pernitrate of mercury, an influence upon the determination of the urea. For instance, if we add 20 C.C. of the graduated mercurial solution to 10 C.C. of a solution of pure urea, then carbonate of soda will produce in the mixture a decided yellow color, of precipitated oxide of mercury. But if we add to the mixture 100—200 milligrms. of chloride of sodium, then try the test, the yellow color does not appear from the addition of carbonate of soda. To effect this, 1½—2½ C.C. more of mercurial solution must be added. On this account the determination would be given too high by 15 to 25 milligrms. Such is the case also with urine; containing more than two per cent. of chloride of sodium, the error does not increase with the increase of the latter, but remains constant with certain fluctuations.*

In a urine, now, that contains from one to one and a half per cent. of chloride of sodium, we can, without anything further, obtain the exact number of milligrms. of urea in 10 C.C. of urine, by subtracting 2 C.C. from the number of C.C. of mercurial solution used, and also, if the amount of chloride of sodium fluctuates with different individuals, then the difference obtained in the amount of urea is exact, and can be compared with others. In the absolute quantity only is there an error, which, uncorrected, would amount to 15—20 milligrms. in 10 C.C. of urine.

* In order to explain this peculiarity, it is evident, from the method of determining chloride of sodium by means of pernitrate of mercury, that a solution of urea containing chloride of sodium will not precipitate by nitrate of mercury, until the former is completely converted into bichloride of mercury. In a solution of 200 milligrms. of urea and 100 milligrms. of chloride of sodium in 10 C.C. of water, to which is added 20 C.C of mercurial solution, the excess of mercurial salt, which should have given the yellow color upon the addition of carbonate of soda, would not exist in the form of a nitrate, but as a bichloride of mercury, and it is evident that the change of the indication of complete precipitation is caused by the formation and presence of this bichloride. Instead of 3.46 milligrms. of oxide of mercury in the form of a nitrate, the mixture contains an equivalent of mercury in the form of bichloride. The reason why the limit of reaction does not extend when the chloride of sodium exceeds two per cent., Liebig explains by the following: If, to a solution of bichloride of mercury, diluted with water to such a degree that it yields with carbonate of soda a brown yellow precipitate of oxide of mercury, be added a few drops of nitric acid, and then carbonate of soda added to it, it will remain clear, no precipitate forms, or at least only a weak whitish cloudiness, out of which, after long standing, brown yellow leaflets will be deposited. In this condition the excess of bichloride of mercury exists in the mixture of urea with the mercurial solution; it contains the greater part of the nitric acid of the latter in free condition. By this nitric acid, a part of the carbonate of soda will be converted into bicarbonate, which does not precipitate the bichloride. If the mixture, in consequence of a greater amount of chloride of sodium, contains a greater amount of bichloride of mercury, then the free carbonic acid is not sufficient to prevent the precipitation of the oxide of mercury; there arises, therefore, a brownish yellow precipitate.

In a determination, in which the absolute quantity of urea is sought for, the chloride must be removed, and the chloride of sodium be converted into nitrate of soda. This takes place by precipitation through nitrate of silver. For this purpose, we must prepare a silver solution from 11.601 grmms. of melted nitrate of silver, dissolve it in water, and dilute it so that the volume of solution consists of 400 C.C. .1 C.C. contains 29.01 milligrms. of nitrate of oxide of silver, corresponding to 10 milligrms. of chloride of sodium. The mercurial solution (the preparation of which, for determining chloride of sodium, has been given above) corresponds with this solution; both exhibit the same amount of chloride of sodium. If we, accordingly, add to 10 C.C. of urine 12.5 C.C. of mercurial solution until the appearance of the cloudiness; in a similar volume of urine, by adding 12.5 C.C. of solution of silver, the chloride will be completely precipitated, and no silver remain in solution.

Suppose we have used to 15 C.C. of urine, precipitated by solution of baryta (containing 10 C.C. of urine,) 17.5 C.C. of mercurial solution, then measure with the pipette,

30 C.C. of the same urine, and add
35 " of silver solution.
—
65

Now filter, and take from the fluid filtered, for the test, half the amount of the mixed fluid—therefore, 32.5 C.C.—in which exists 10 C.C. of urine. This is now mixed with the graduated mercurial solution, and the amount of urea obtained in the manner described, regard being paid to the dilution in consequence of the silver solution that has been added.—*Annalen der Chemie und Pharmacie*, 85. *Bd.* 3, *Heft. page* 289. 1853.

NOTE.—The reader should bear in mind, that the only difficulty in the above processes consists in the first preparation of the test liquids. If they are once correctly made, and in sufficient quantity to last through a series of experiments, each examination of urine can be made with great accuracy, in a very few minutes, and without much exertion of skill, on the part of the operator.—*Translator.*

